# Lack of association between IGF2BP2 rs4402960 polymorphism and gestational diabetes mellitus: a case–control study, meta-analysis and trial sequential analysis

**DOI:** 10.1042/BSR20200990

**Published:** 2020-07-23

**Authors:** Jing Liu, Guang Song, Ge Zhao, Tao Meng

**Affiliations:** 1 Department of Obstetrics, The First Affiliated Hospital of China Medical University, Shenyang, China; 2 Department of Ultrasound, Shengjing Hospital of China Medical University, Shenyang, China

**Keywords:** Genetic variation, Gestational diabetes mellitus, Meta-analysis, Polymorphisms

## Abstract

**Background:** It is well known that insulin-like growth factor 2 mRNA-binding protein 2 (*IGF2BP2*) rs4402960 polymorphism is associated with Type 2 diabetes mellitus, which has a shared genetic background with gestational diabetes mellitus (GDM). Previous studies have yielded controversial results about the link between *IGF2BP2* rs4402960 polymorphism and GDM risk. Thus, a meta-analysis was performed to obtain more conclusive results.

**Methods**: Clinical and genotype data were determined for 305 GDM and 1216 healthy participants recruited. Eligible studies were retrieved in PubMed, Web of science, EMBASE, and Scopus. Odds ratios (ORs) with 95% confidence intervals (CIs) were utilized to evaluate the relationship between *IGF2BP2* polymorphisms and GDM susceptibility in five genetic models. The subgroup stratified analysis and trial sequential analysis (TSA) were performed.

**Results**: In this case–control study, no significant association was revealed between *IGF2BP2* polymorphism and GDM (*P*>0.05). When combined with the previous studies in the meta-analysis, there was no statistical association between *IGF2BP2* polymorphism and GDM (allele model: OR = 1.01, 95% CI = 0.86–1.18; dominant model: OR = 1.00, 95% CI = 0.81–1.24; recessive model: OR = 1.08, 95% CI = 0.91–1.29; heterozygous model: OR = 0.99, 95% CI = 0.80–1.24; homozygous model: OR = 1.06, 95% CI = 0.78–1.42). No association was observed in five genetic models in each subgroup. TSA indicated sufficient proof of such null association in the overall population.

**Conclusions**: This meta-analysis provides sufficient statistical evidence indicating null association between *IGF2BP2* rs4402960 polymorphism and GDM risk.

## Introduction

Gestational diabetes mellitus (GDM) is defined as glucose intolerance that occurs during pregnancy. The GDM is the most prevalent metabolic disorder during pregnancy with prevalence between 9.3% and 25.5%, which is attributed mainly to obesity, older maternal age, and a sedentary lifestyle [1]. GDM results in significant perinatal mortalities and comorbidities. Pregnant women complicated with GDM tend to have increasing risk of obesity, diabetes mellitus (DM), and cardiovascular disease. The underlying factors leading to the development of GDM are difficult to determine, and may involve a combination of diverse environmental, genetic, and epigenetic factors. An increasing amount of evidence points to a connection between genetics and GDM [2].

Insulin-like growth factor 2 mRNA-binding protein 2 (*IGF2BP2*) is part of the family of mRNA-binding proteins regulating insulin-like growth factor 2 (*IGF2*) translation. *IGF2BP2* is located on chromosome 3q27. A previous animal study showed that *IGF2* is involved in pancreatic function [3]. Several studies have reported that the polymorphisms in *IGF2BP2* is associated with reduced β-cell function [4,5] and risk of DM [6]. Previous studies revealed that 2.6–70% of women with a history of GDM will develop DM 28 years postpartum [7], and women with a family history of DM may be predisposed to an increased risk of GDM [8]. GDM may share the same risk factors and genetic susceptibilities with DM that indicates that *IGF2BP2* gene polymorphism may also be associated with GDM.

To date, researchers investigated the association between three *IGF2BP2* polymorphisms (rs4402960, rs1470579, rs11705701) and GDM. There was no evidence of the association between *IGF2BP2* rs1470579 or rs11705701 and risk of GDM [9,10]. Recently, the association between *IGF2BP2* rs4402960 polymorphism and risk of GDM has been discussed in many studies [11,12]. However, the results were inconsistent. Therefore, we conducted a meta-analysis of studies on the *IGF2BP2* polymorphism with the aim of providing a more comprehensive summary of currently available research to evaluate the relationship between the *IGF2BP2* rs4402960 polymorphism and GDM risk.

## Methods

### Study subjects

Between January 2014 and December 2018, 1521 singleton pregnant women were enrolled in the present study in the Department of Obstetrics, at the First Affiliated Hospital of China Medical University. All participants were provided with written informed consent and the study protocol was approved by the Medical Ethics Review Board of China Medical University (Shenyang, Liaoning, China).

Included GDM cases were identified after a glucose challenge test between weeks 24 and 28 of gestation. Participants were not eligible if they had a history of diabetes (including GDM in previous pregnancies). The control group consisted of 1216 middle-aged (18–45 years) non-diabetic Chinese women from the prospective hospital-based cohort [13]. We excluded those who presenting with maternal-related abnormalities, mothers with a history of drug abuse and depression, carriers of a blood-transmitted infectious disease, hypertension, and cardiovascular diseases.

All pregnant women were screened for GDM at 24–28 gestation weeks with a 75 g, 2 h oral glucose tolerance test. GDM was defined according to the International Association of Diabetes and Pregnancy Study Groups [14]. A diagnosis of GDM was made when one or more of the test parameters equaled or exceeded the following cut points: fasting: 5.1 mmol/l, 1-h: 10.0 mmol/l, or 2-h: 8.5 mmol/l. We also measured anthropometric parameters such as weight and height. Body mass index (BMI) was calculated. We also recorded maternal age, gravida, parity, family history of diabetes, and lipid profile.

### Genotyping

About 5 ml blood sample was collected from each participant. DNA was extracted with standard proteinase K digestion, followed by phenol–chloroform extraction and ethanol precipitation.

Genotyping of IGF2BP2 rs4402960 was performed with the Taqman allelic discrimination assays using ABI 7500 Real Time PCR (Applied Biosystems, Foster City, CA). All primers and probes were designed by Applied Biosystems (Foster City, CA). The primers sequence were 5′-GGAGCAGTAAGGTAGGATGGACAGTAGATT-3′ (forward) and 5′-AAGATACTGATTGTG TTTGCAAACATGCCC-3′ (reverse). Assay ID is C__2165199_10, and context sequence is AGTAAGGTAGGATGGACAGTAGATT[G/T]AAGATACTGATTGTGTTTGCAAACA. The reaction mix included 10 μl DNA, 25 μl master mix (Applied Biosystems), 2.5 μl probe, and 12.5 μl sterile water in a final volume of 50 μl. PCR conditions comprised an initial denaturing step at 95°C for 10 min, followed by 45 cycles of 92°C for 30 s, with a final extension at 60°C for 1 min. PCR plates were read on an ABI PRISM 7900 instrument (Applied Biosystems). Two authors (G.S. and G.Z.) independently reviewed the genotyping results and input data.

### Meta-analysis

Two examiners (J.L. and G.S.) independently searched for the significant studies in databases, including PubMed, Web of science, EMBASE, and Scopus on March 15, 2020. The search terms are the ‘gestational diabetes mellitus’, ‘polymorphism/variant/mutation/’, and ‘*IGF2BP2’*. Original studies were eligible if they met the following criteria: (I) case-control studies; (II) literatures on associations between *IGF2BP2* rs4402960 polymorphism and GDM; (III) results were denoted as odds ratio (OR) with 95% confidence intervals (CI). Original studies were ineligible if they: (I) were reviews, letters, or case reports; (II) did not contain outcomes which can be converted to OR and 95% CI; (III) included control group that was not involved with healthy women; (IV) were laboratory studies with animals. For duplicate publications, we only included the study with the largest sample size for analyses.

Two researchers (J.L. and G.Z.) carried out data extraction independently. If there were disagreements, they discussed and reached a consensus with the help from a third reviewer (TM). The data contained the first author, publication year, ethnicity, control source, genotyping method, genotype distribution in case and control groups. The Newcastle–Ottawa Scale (NOS) was used to assess the quality of all studies included. The NOS quality score ranges from 0 to 9 stars. Two authors independently assessed the quality of the studies included. Disagreements were resolved by discussion.

### Statistical analysis

Chi-square test was used to compare Allele/genotype frequencies between GDM and control groups. The strength of association between *IGF2BP2* rs4402960 polymorphism and GDM risk was assessed by calculating the OR with 95% CI. We calculated the OR by genotype and allele model comparisons of *IGF2BP2* rs4402960 polymorphism between cases and controls. The *P* value for Hardy–Weinberg equilibrium (HWE) was calculated in the control group using the chi-square test. Heterogeneity was assessed by using the *I*^2^ statistics. If there was no heterogeneity (*P*>0.1 or *I*^2^<50%), a fixed-effects model was used to estimate the pooled OR; otherwise, a random-effects model was utilized. Subgroup stratified analysis was performed using genotyping method (TaqMan assay or PCR), ethnicity (Caucasian or Asian), and control source (hospital-based or population-based). Study effects, such as publication bias, were evaluated with Egger’s tests and a *P* value of < 0.1 was considered statistically significant for asymmetry. Sensitivity analyses were directed to assess the influence of individual study on the overall estimate. Statistical analyses were performed with Stata (version 14.0; StataCorp, College Station, TX). The TSA (version 0.9.5.10, http://www.ctu.dk/tsa/) was conducted to maintain a 95% confidence interval, a 20% relative risk reduction, overall 5% risk of a type I error and 20% of the type II error (a power of 80%) in this study to reduce the risk of type I error.

## Results

### Case–control study

In total, 305 patients with GDM and 1216 controls participated in the present study. The baselines of characteristics of participants are shown in Table 1. Compared to the control groups, there was no difference in the maternal age, height, weight, pre-pregnancy BMI, gravida, parity, family history of diabetes, and low-density lipoprotein cholesterol (*P*>0.05). There were significant differences between the two groups with respect to triglyceride, total cholesterol, and high-density lipoprotein cholesterol (*P*<0.05). There was no difference in genotype frequencies between GDM and controls groups in *IGF2BP2*. Meanwhile, there was no significant susceptibility for rs4402960 polymorphism with GDM risk within five genetic models (all *P*>0.05; Table 2).

**Table 1:** Table 1 Clinical characteristics of pregnant women enrolled in the study

	GDM (*n*=305)	Control (*n*=1216)	*P*
Maternal age (years)	32.13 ± 3.04	32.13 ± 3.04	0.985
Height (m)	1.63 ± 0.04	1.63 ± 0.05	0.303
Weight (kg)	60.4 ± 7.24	59.8 ± 7.63	0.195
Pre-pregnancy BMI (kg/m2)	22.85 ± 3.06	22.69 ± 3.08	0.417
Gravida	1 (1–2)	1 (1–2)	0.370
Parity	0 (0–1)	0 (0–1)	0.994
Family history of diabetes	20 (7%)	64 (5%)	0.376
Triglyceride (mg/dl)*	232.53 ± 61.48	223.83 ± 62.16	0.029
Total cholesterol (mg/dl)*	241.06 ± 29.44	236.87 ± 30.67	0.032
High-density lipoprotein cholesterol (mg/dl)*	67.98 ± 8.93	69.18 ± 7.09	0.013
Low-density lipoprotein cholesterol (mg/dl)*	129.35 ± 22.02	131.44 ± 22.19	0.141

BMI, body mass index.

Maternal age, height, weight, and pre-pregnancy BMI were expressed as the mean ± standard deviation.

Gravida and parity were expressed as the median and interquartile ranges (interquartile ranges: the range of values lying between 25th and 75th centiles.

* : test from each participant before 19 weeks + 6 days.

**Table 2:** Genotype frequencies and analysis of *IGF2BP2* rs4402960 in Chinese population

	*n* (%)		*P*	Comparison	Type of model	OR (95% CI)	*P*
	GDM	Control group					
GG	168 (55%)	608 (50%)	0.179	T vs. G allele	Allele	0.83 (0.68–1.01)	0.060
GT	115 (38%)	488 (40%)		TT+GT vs. GG	Dominant	0.82 (0.63–1.05)	0.112
TT	22 (7%)	120 (10%)		TT vs. GG+GT	Recessive	0.71 (0.44–1.14)	0.154
				TT vs. GT	Heterozygous	0.85 (0.65–1.11)	0.322
				TT vs. GG	Homozygous	0.66 (0.41–1.08)	0.096

Abbreviations: CI, confidence interval; OR, odds ratio.

### Literature search

**Figure 1:**
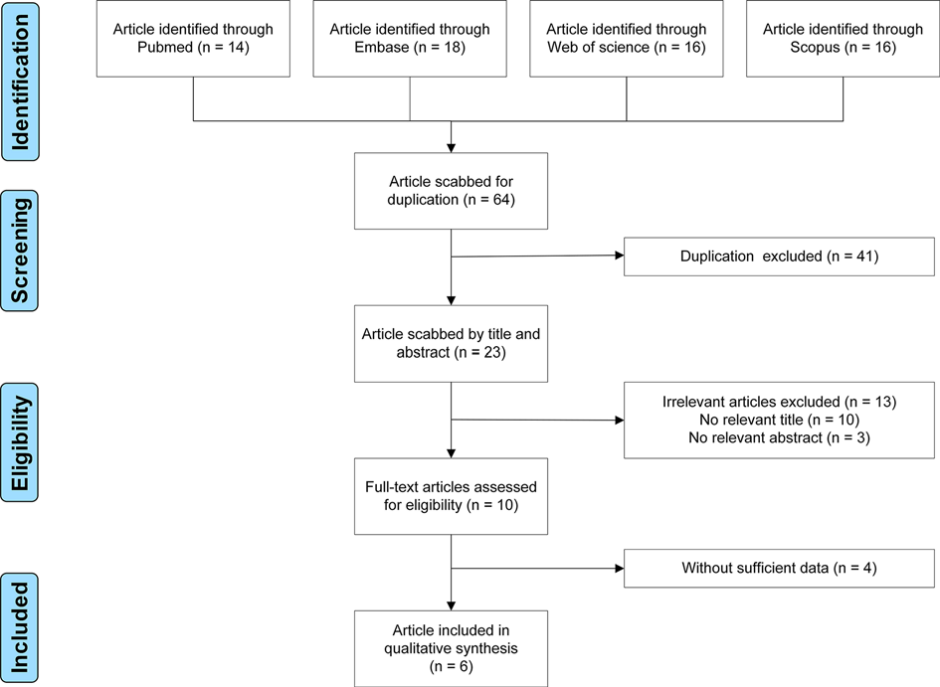
Flow chart of study selection

About 64 potentially eligible studies were retrieved. After application of the criteria, six studies were kept (Figure 1). Characteristics of the seven studies, including ours, are shown in Table 3. The published year ranged from 2009 to 2020. The subjects in four were Asians, and Caucasians in three studies. Six were hospital-based design, only one was population-based design. Five studies used TaqMan assay, and two studies used polymerase chain reaction-restriction fragment length polymorphism (PCR-RFLP). NOS scores were between six and eight (details are shown in the **Supplementary Material**). A total of 8346 subjects were involved, including 2720 GDM patients and 5626 healthy women. All *P* values of HWE were more than 0.05.

**Table 3:** Characteristics of 2720 gestational diabetes mellitus cases and 5626 controls included in this meta-analysis

First author	Year	Country	Control source	Genotyping method	NOS Score	Case (*n*)				Control (*n*)				HWE (*P*)
						Total	GG	GT	TT	Total	GG	GT	TT	
Lauenborg	2009	Denmark	Hospital-based	TaqMan assay	6	274	115	132	27	2334	1138	972	224	0.433
Cho	2009	Korea	Population-based	TaqMan assay	6	857	389	365	103	627	313	257	57	0.685
Wang	2011	China	Hospital-based	TaqMan assay	8	705	371	278	56	1025	605	361	59	0.596
Chon	2013	Korea	Hospital-based	PCR-RFLP	6	94	57	30	7	41	15	24	2	0.053
Popova	2017	Russia	Hospital-based	PCR-RFLP	7	278	120	134	24	179	77	76	26	0.310
Tarnowski	2019	Poland	Hospital-based	TaqMan assay	7	207	105	76	26	204	89	93	22	0.753
Current study	2020	China	Hospital-based	TaqMan assay	6	305	168	115	22	1216	608	488	120	0.131

Abbreviations: HWE, Hardy–Weinberg equilibrium; NOS, Newcastle–Ottawa Scale; PCR-RFLP, polymerase chain reaction-restriction fragment length polymorphism.

### Quantitative synthesis

**Figure 2:**
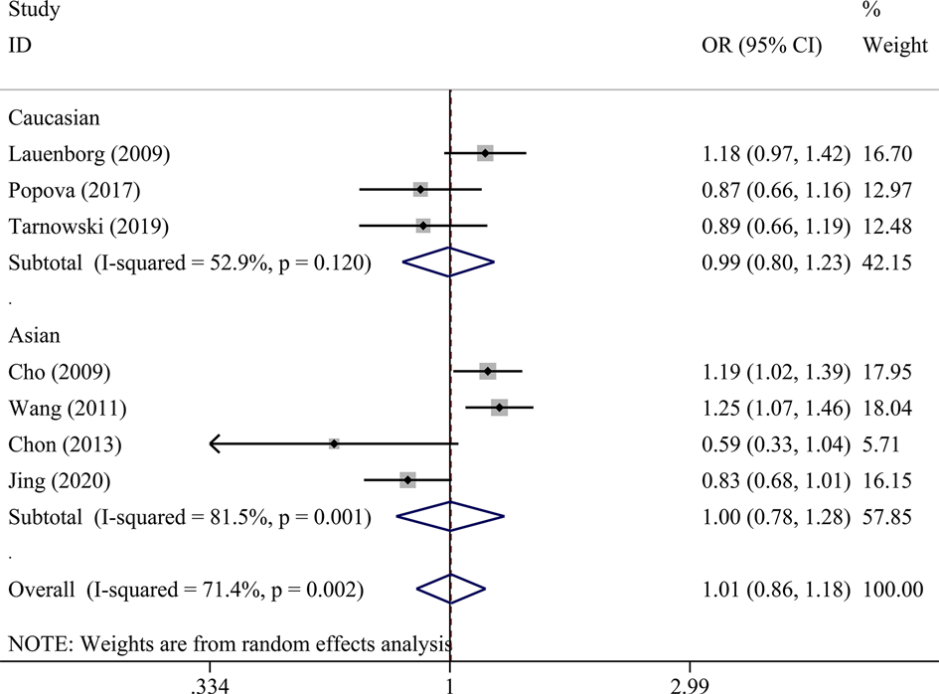
Forest plot for the association between *IGF2BP2* rs4402960 polymorphism and GDM risk using ethnicity subgroup analysis in the allele model (T vs. G allele)

**Figure 3:**
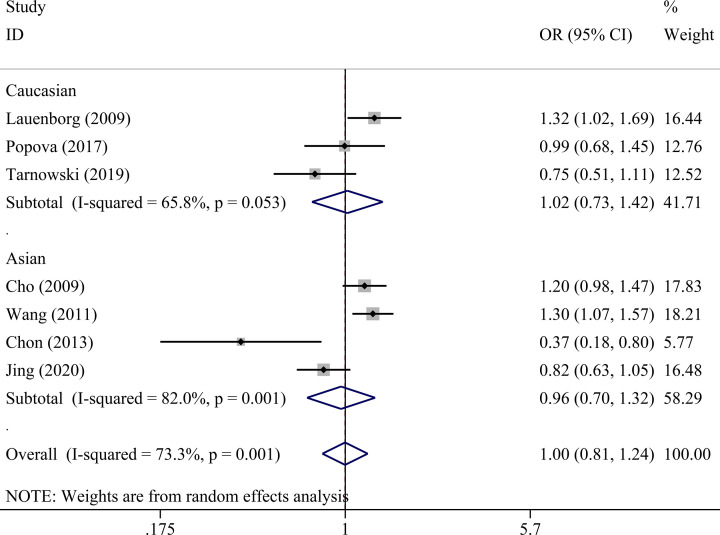
Forest plot for the association between *IGF2BP2* rs4402960 polymorphism and GDM risk using ethnicity subgroup analysis in the dominant model (TT+GT vs. GG)

**Figure 4:**
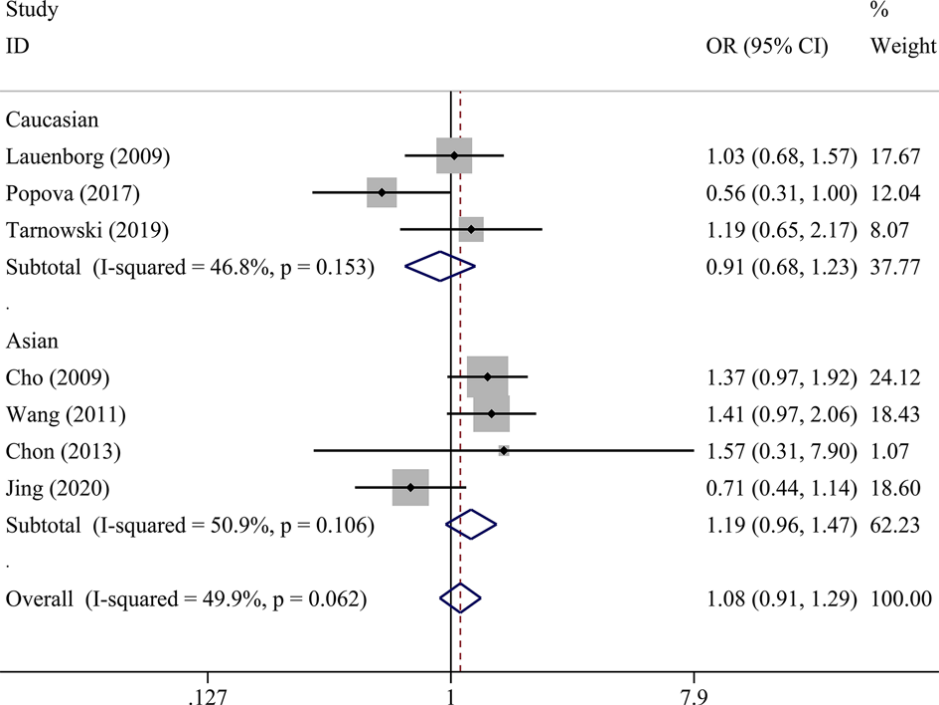
Forest plot for the association between *IGF2BP2* rs4402960 polymorphism and GDM risk using ethnicity subgroup analysis in the recessive model (TT vs. GG+GT)

**Figure 5:**
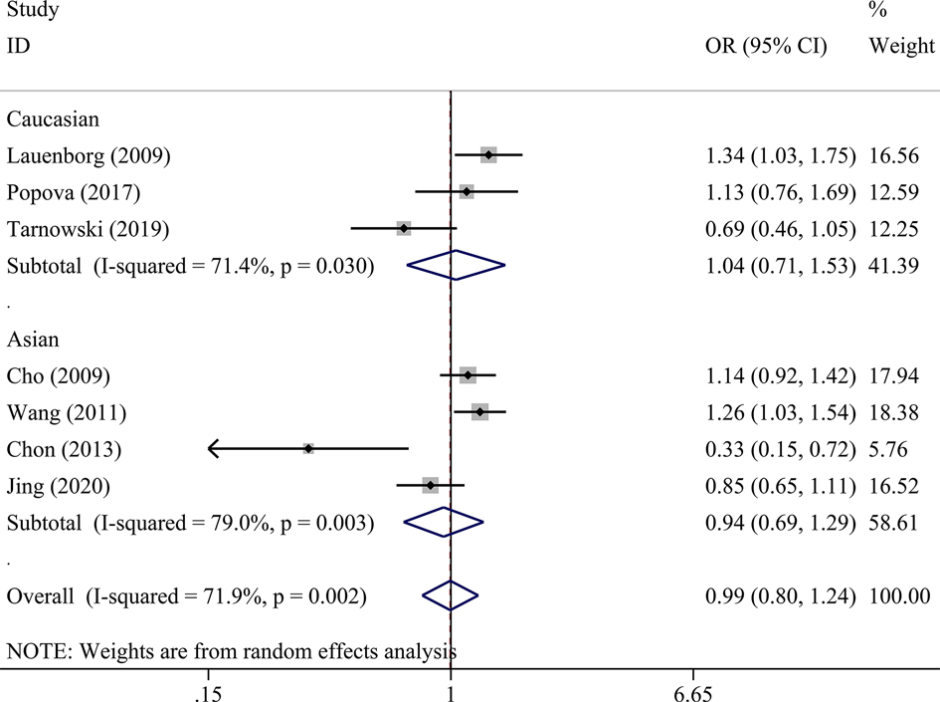
Forest plot for the association between *IGF2BP2* rs4402960 polymorphism and GDM risk using ethnicity subgroup analysis in the heterozygous model (TT vs. GT)

**Figure 6:**
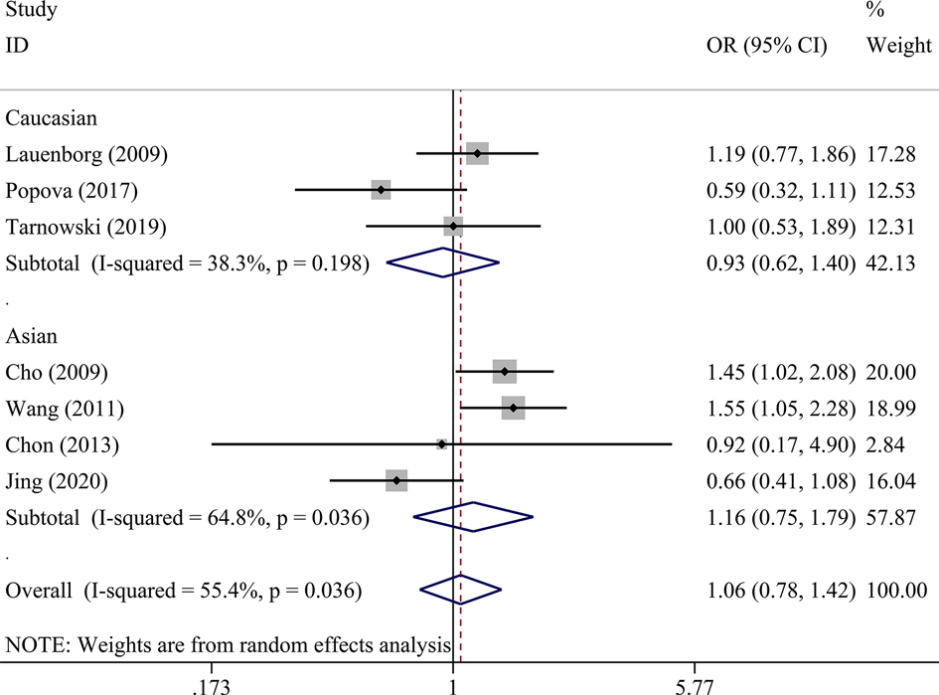
Forest plot for the association between *IGF2BP2* rs4402960 polymorphism and GDM risk using ethnicity subgroup analysis in the homozygous model (TT vs. GG)

There was no statistically significant association between *IGF2BP2* rs4402960 polymorphism and GDM within five genetic models as shown in Table 4: allele (OR = 1.01, 95% CI = 0.86–1.18), dominant (OR = 1.00, 95% CI = 0.81–1.24), recessive (OR = 1.08, 95% CI = 0.91–1.29), heterozygous (OR = 0.99, 95% CI = 0.80–1.24), and homozygous models (OR = 1.06, 95% CI = 0.78–1.42). Subgroup analysis was conducted to reveal some details regarding potential associations between *IGF2BP2* rs4402960 polymorphism and GDM risk. No association was observed in five genetic models in each subgroup (Table 5 and Figures 2–6).

**Table 4:** Meta-analysis of association between *IGF2BP2* rs4402960 polymorphisms and risk of gestational diabetes mellitus

Comparison	Type of model	Test of association		Test of heterogeneity		Statistical model	Test of publication bias Egger's, *P* value	Sensitivity analysis	
		Odds ratio (95% CI)	*P*	*I*^2^ (%)	*P*			Odds ratio (95% CI) _min_	Odds ratio (95% CI) _max_
T vs. G allele	Allele	1.01 (0.86–1.18)	0.950	71.4	0.002	Random	0.072	0.96 (0.80–1.14)	1.06 (0.91–1.23)
TT+GT vs. GG	Dominant	1.00 (0.81–1.24)	0.986	73.3	0.001	Random	0.072	0.94 (0.73–1.20)	1.07 (0.89–1.28)
TT vs. GG+GT	Recessive	1.08 (0.91–1.29)	0.368	49.9	0.062	Fixed	0.368	0.99 (0.81–1.22)	1.17 (0.97–1.41)
TT vs. GT	Heterozygous	0.99 (0.80–1.24)	0.964	71.9	0.002	Random	0.055	0.93 (0.72–1.20)	1.08 (0.90–1.28)
TT vs. GG	Homozygous	1.06 (0.78–1.42)	0.718	55.4	0.036	Random	0.368	0.97 (0.70–1.34)	1.18 (0.90–1.55)

Abbreviations: CI, confidence interval; OR, odds ratio.

**Table 5:** Subgroup analysis of the associations of *IGF2BP2* rs4402960 (G>T) polymorphisms with gestational diabetes mellitus

Subgroup	*N*	Allele model (T vs. G allele)				Dominant model (TT+GT vs. GG)				Recessive model (TT vs. GG+GT)				Heterozygous model (TT vs. GT)				Homozygous model (TT vs. GG)			
		Test of association		Test of heterogeneity		Test of association		Test of heterogeneity		Test of association		Test of heterogeneity		Test of association		Test of heterogeneity		Test of association		Test of heterogeneity	
		Odds ratio (95% CI)	*P*	*I*^2^ (%)	*P*	Odds ratio (95% CI)	*P*	*I*^2^ (%)	*P*	Odds ratio (95% CI)	*P*	*I*^2^ (%)	*P*	Odds ratio (95% CI)	*P*	*I*^2^ (%)	*P*	Odds ratio (95% CI)	*P*	*I*^2^ (%)	*P*
Genotyping method																					
TaqMan assay	5	1.07 (0.91–1.26)	0.410	71.7	0.007	1.08 (0.87–1.33)	0.491	72.0	0.006	1.15 (0.96–1.38)	0.135	37.3	0.173	1.06 (0.87–1.30)	0.552	67.4	0.015	1.16 (0.86–1.56)	0.321	54.6	0.066
PCR-RFLP	2	0.78 (0.55–1.11)	0.164	33.1	0.222	0.65 (0.25–1.67)	0.368	80.4	0.024	0.64 (0.37–1.10)	0.107	28.6	0.237	0.64 (0.19–2.14)	0.468	86.8	0.006	0.63 (0.35–1.12)	0.115	0	0.628
Ethnicity																					
Caucasian	3	0.99 (0.80–1.23)	0.949	52.9	0.120	1.02 (0.73–1.42)	0.906	65.8	0.053	0.91 (0.68–1.23)	0.550	46.8	0.153	1.04 (0.71–1.53)	0.837	71.4	0.030	0.93 (0.62–1.40)	0.722	38.3	0.198
Asian	4	1.00 (0.78–1.28)	0.988	81.5	0.001	0.96 (0.70–1.32)	0.810	82.0	0.001	1.19 (0.96–1.47)	0.120	50.9	0.106	0.94 (0.69–1.29)	0.722	79.0	0.003	1.16 (0.75–1.79)	0.510	64.8	0.036
Control source																					
Hospital-based	6	0.96 (0.80–1.16)	0.691	73.4	0.002	0.94 (0.72–1.23)	0.675	76.7	0.001	0.99 (0.80–1.22)	0.953	48.2	0.086	0.95 (0.92–1.25)	0.703	76.3	0.001	0.97 (0.69–1.37)	0.874	54.0	0.054
Population-based	1	1.19 (1.02–1.39)	0.031	–	–	1.20 (0.98–1.47)	0.084	–	–	1.37 (0.97–1.92)	0.073	–	–	1.14 (0.92–1.42)	0.231	–	–	1.45 (1.02–2.08)	0.039	–	–

Abbreviations: CI, confidence interval; HWE, Hardy–Weinberg equilibrium; OR, odds ratio; PCR-RFLP, polymerase chain reaction-restriction fragment length polymorphism.

### Sensitivity analysis and publication bias

There was no change after the deletion of any of the studies involved in the investigation, and no publication bias was detected (Table 4).

### Trial sequential analysis

**Figure 7:**
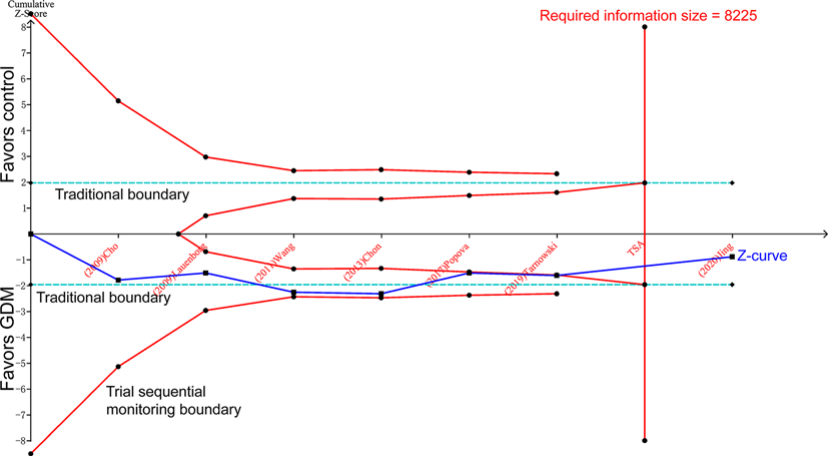
Trial sequential analysis of GDM risk associated with *IGF2BP2* rs4402960 polymorphism in the recessive model

The cumulative *Z*-curve passed both the traditional boundary and required information size line, indicating sufficient proof of such association within the overall population (Figure 7).

## Discussion

Our case–control study did not reveal any association between *IGF2BP2* rs4402960 polymorphism and GDM risk. Further meta-analysis involving seven studies indicated that *IGF2BP2* rs4402960 polymorphism was not associated with GDM risk in overall population, Caucasian, or Asian under various genetic models. TSA analysis indicated that the pooled sample size was sufficient to support these null associations.

*IGF2BP2* belongs to a mRNA-binding protein family that plays important roles in RNA localization, stability, and translation [15]. *IGF2BP2* is highly expressed in pancreatic islets and binds to *IGF2*, which is an important growth and insulin signaling molecule [15]. Previous studies have shown the involvement of *IGF2BP2* gene polymorphism in attenuating the first phase of glucose-stimulated insulin secretion based on hyperglycaemic clamps, and the regulation of pancreatic β-cell function in Type 2 diabetes mellitus (T2DM) patients [16]. Therefore, the *IGF2BP2* rs4402960 polymorphism plays a role in pathogenesis of T2DM [17]. T2DM and GDM share a similar genetic background due to the common family history and similar features of impaired glucose tolerance between these two diseases [18]. Several T2DM-associated genetic variants have been confirmed to be associated with GDM, such as *TCF7L2* rs12255372 and *MTNR1B* rs10830963 [18] while the effects of other T2DM-associated genetic variants on GDM are controversial, such as *WFS1* rs10010131 and *ABCA1* rs1800977 [11,19].

Early studies showed that *IGF2BP2* rs4402960 polymorphism was associated with GDM risk [11,12,20]. This finding has been cited in previously published reports [18,21]. However, recent studies revealed there was no association between *IGF2BP2* rs4402960 polymorphism with GDM [10,22]. Results reported to date are inconsistent. This could be due to limited statistical power with individual studies having relatively small sample sizes and the analysis of a specific ethnicity. For many of these studies, meta-analysis was usually performed in terms of genetic association studies of complicated diseases. Therefore, to further investigate the influence of *IGF2BP2* rs4402960 polymorphism on GDM risk, a meta-analysis of six previously published reports in combination with the present results of the case–control study was conducted. As far as we know, this is the first meta-analysis to date comprehensively quantified the association between the *IGF2BP2* and GDM risk.

However, meta-analysis has its potential limitation, resulting in spuriously overestimated (type I errors) or spuriously underestimated (type II errors) effects [23]. As a complement of meta-analysis, TSA was designed. TSA could reduce the risk of type I error by estimation of required information size with an adjusted threshold for statistical significance, and estimate whether further additional trials are needed [24]. If the cumulative *Z*-curve touches the trial sequential monitoring boundary, the futility boundary, or the required information size line, it shows firm evidence for such study. If not, additional studies are necessary to reach a consistent conclusion [24].

In our TSA results, the cumulative *Z*-curve crosses the traditional boundary using the Cho, Lauenborg, Wang, and Chon’s studies [11,12,20,25]. However, sample size of these four studies is not large enough. Two former meta-analyses ended up with the false positive conclusion due to the earlier publish time with insufficient sample size [21,26]. Utilizing the data reported in recent studies, the cumulative *Z*-curve goes back to the zone between two traditional boundaries, then crosses the futility boundary and reaches the required information size line. Until now, there is sufficient evidence to support the null association between *IGF2BP2* rs4402960 polymorphism and GDM risk. This trend of cumulative *Z*-curve explains the TSA advantage, which helps us avoid reaching a premature statistical significance conclusion [23].

### Limitation

There were limitations in the present study. First, the sample size in each subgroup was small. Second, there was no study that includes the Blacks or Hispanics.

## Conclusion

This meta-analysis provides sufficient statistical evidence indicating null association between *IGF2BP2* rs4402960 polymorphism and GDM risk.

## Supplementary Material

Supplementary Table

## Abbreviations

BMI: body mass index
CI: confidence interval
DM: diabetes mellitus
GDM: gestational diabetes mellitus
HWE: Hardy–Weinberg equilibrium
*IGF2BP2*: insulin-like growth factor 2 mRNA-binding protein 2
NOS: Newcastle–Ottawa Scale
OR: odds ratio
PCR-RFLP: polymerase chain reaction-restriction fragment length polymorphism
T2DM: Type 2 diabetes mellitus
