# Supplementary material for: Lack of association between IGF2BP2 rs4402960 polymorphism and gestational diabetes mellitus: a case–control study, meta-analysis and trial sequential analysis
Source: Biosci Rep. 2020 Jul 23;40(7):BSR20200990. doi: 10.1042/BSR20200990 (PMC7378266; doi:10.1042/BSR20200990)
Supplement: Supplementary Table [file BSR-2020-0990_supp.pdf]

Table S1 Summary NOS of the studies included in the meta-analysis

Study	Selection (4 stars)				Comparability (2 stars)	Exposure (3 stars)			Overall
	Is the case definition adequate	Representativeness of the cases	Selection of Controls	Definition of Controls		Ascertainment of exposure	Same method of ascertainment for cases and controls	Non-Response rate	
**Lauenborg (2009)**	★	★	★	★		★	★		6
**Cho (2009)**	★	★	★	★		★	★		6
**Wang (2011)**	★	★	★	★	★	★	★	★	8
**Chon (2013)**	★	★	★	★	★		★		6
**Popova (2017)**	★	★	★	★		★	★	★	7
**Tarnowski (2019)**	★	★	★	★		★	★	★	7
**Current study**	★	★	★	★		★	★		6
